# Pancreatico-Colonic Fistula: A Case Report on Diagnosis and Management

**DOI:** 10.7759/cureus.96012

**Published:** 2025-11-03

**Authors:** Geuel Simiyu, Matthew J Li, Mujahed Laswi, LaJohn Quigley, Izi Obokhare

**Affiliations:** 1 Department of Surgery, Texas Tech University Health Sciences Center, Amarillo, USA

**Keywords:** acute pancreatitis, diagnosis, management, necrotizing pancreatitis, pancreatico-colonic fistula

## Abstract

Pancreatico-enteric fistulas are rare complications of chronic pancreatitis, with colonic involvement carrying particularly high morbidity and mortality. Early recognition and prompt surgical management are critical to improving outcomes. We present a case of a patient with necrotizing pancreatitis who developed a pancreatico-colonic fistula five months following initial hospitalization.

A male in his 40s with a history of poorly controlled diabetes, hypertension, and gallstone pancreatitis (status post-cholecystectomy) was transferred to our tertiary care hospital in septic shock. Imaging revealed necrotizing pancreatitis with a splenic artery pseudoaneurysm, which was successfully embolized. CT-guided drainage of peripancreatic collections grew *Escherichia (E.) coli* (extended-spectrum beta-lactamase or ESBL), vancomycin-resistant Enterococcus (VRE), and *Streptococcus viridans*, requiring broad-spectrum antibiotics. The patient improved and was discharged after one month, but was lost to follow-up. Five months later, he was readmitted with persistent *Streptococcus* bacteremia. CT abdomen/pelvis with rectal contrast confirmed a pancreatico-colonic fistula at the splenic flexure. He underwent a robotic-assisted left colectomy with takedown of the fistula and end colostomy. Postoperatively, he recovered well, tolerated diet advancement, and was discharged on postoperative day seven.

Pancreatico-colonic fistulas often result from spontaneous decompression of a pancreatic pseudocyst or abscess into adjacent bowel loops. Unlike other pancreatico-enteric fistulas, colonic involvement carries a significant risk due to the translocation of colonic flora and potential for sepsis. While small fistulas may respond to endoscopic closure, surgical resection remains the standard of care in patients with sepsis, large defects, or multiple perforations.

Although uncommon, pancreatico-colonic fistula should be considered in patients with recurrent sepsis or bacteremia and a history of severe or necrotizing pancreatitis. Early imaging, surgical consultation, and definitive operative management are essential to improve outcomes.

## Introduction

Acute pancreatitis is an inflammatory disorder of the pancreas. The most common etiologies are gallstones (21-33%) and alcohol misuse (16-27%) [1]. In recent years, acute pancreatitis has become one of the leading causes of gastrointestinal disorder-related admissions in United States hospitals [2]. The risk of developing chronic pancreatitis is 13% within 10 years of the initial presentation of acute pancreatitis [3]. An exceedingly uncommon complication seen with chronic pancreatitis is pancreatico-enteric fistula formation [4]. Fistulation has been reported to the stomach, duodenum, small intestine, colon, and biliary tract [5]. The transverse colon and the splenic flexure of the colon appear to be the most commonly involved sites, with an incidence of 3-10% in patients with chronic pancreatitis. It is thought that fistula formation is the result of spontaneous decompression of an existing pancreatic abscess or pseudocyst into an adjacent organ [6]. The released activated lytic pancreatic enzymes digest the colonic wall, leading to the subsequent formation of a fistula [5]. We present the diagnosis and treatment of a patient with necrotizing pancreatitis who developed a pancreatico-colonic fistula after a lack of proper follow-up after an interventional radiology (IR) drain placement.

## Case presentation

Presentation

The patient is a male in his 40s with a past medical history of severely uncontrolled type 2 diabetes mellitus (T2DM), hypertension, a complicated history of gallstone pancreatitis, and a surgical history of a cholecystectomy. The patient initially presented to our tertiary hospital as a transfer for septic shock, where he was admitted to the Medical Intensive Care Unit (MICU) with a one-day history of gastrointestinal (GI) bleeding with hematemesis and melena. On presentation, he was tachycardic (113 bpm), tachypneic (40/min), hypotensive (85/61 mmHg), fever of 104 °F, a white blood cell count (WBC) of 19.0K/uL, a lactic acid of 5.4, and a hemoglobin/hematocrit (H/H) of 9.3 g/dL/27%. The patient was given one unit of packed red blood cells (PRBC) and four units of fresh frozen plasma (FFP) due to presenting vitals, hematemesis, low H/H levels, and a history of recurrent GI bleeds.

Diagnostic assessment

A CT scan of the abdomen and pelvis (CT AB/P) without contrast showed a large multiloculated, left-sided retroperitoneal air-fluid collection involving the peripancreatic soft tissues with trace complex ascites (concerning for either necrotizing pancreatitis or perforated viscus), hepatic steatosis, and hepatomegaly. He was then started on prophylactic vancomycin, Zosyn, pantoprazole, and metronidazole for the suspected infection, and GI PCR, stool culture, and *Clostridium difficile (C. diff)* were collected. General surgery was consulted and suggested a CT angiogram (CTA) with IV contrast of the abdomen and pelvis, which confirmed the diagnosis of necrotizing pancreatitis and located active bleeding within the peripancreatic collection, potentially from the splenic artery. He subsequently underwent emergent selective splenic artery embolization accessed from the right common femoral artery. Angiography during the procedure confirmed a pseudoaneurysm in the splenic artery with mild bleeding. Stagnant flow in the distal artery was confirmed post-embolization with injection of IV contrast proximal to the occluding coils. Repeat CT post-embolization showed no evidence of rebleeding or gastric perforation. The following day, he underwent CT-guided drainage of air-fluid collections, producing approximately 15 cc of dark bloody fluid from the left upper quadrant (LUQ) perisplenic collection and 60 cc of dark bloody fluid from the left lower quadrant (LLQ) retroperitoneal collection. Pigtail drains were left at these locations to continue fluid drainage. GI PCR, stool culture, and *C. diff* cultures came back negative. Cultures of collected retroperitoneal fluid grew *Escherichia coli (E. Coli)* producing extended-spectrum beta-lactamases (ESBL), vancomycin-resistant Enterococcus (VRE) faecium, and *Streptococcus viridans*. As a result, IV antibiotics were transitioned to meropenem, linezolid, and fluconazole. On hospital day 4, the patient was moved to floor status due to a stable hemoglobin and improvement of symptoms. Continued treatment of sepsis was managed by internal medicine, and the patient was discharged after one month inpatient. He received pneumococcal, meningococcal, and *Haemophilus influenzae* vaccines and was scheduled to follow up with surgery in two weeks. However, the patient did not attend his follow-up visit.

**Figure 1:**
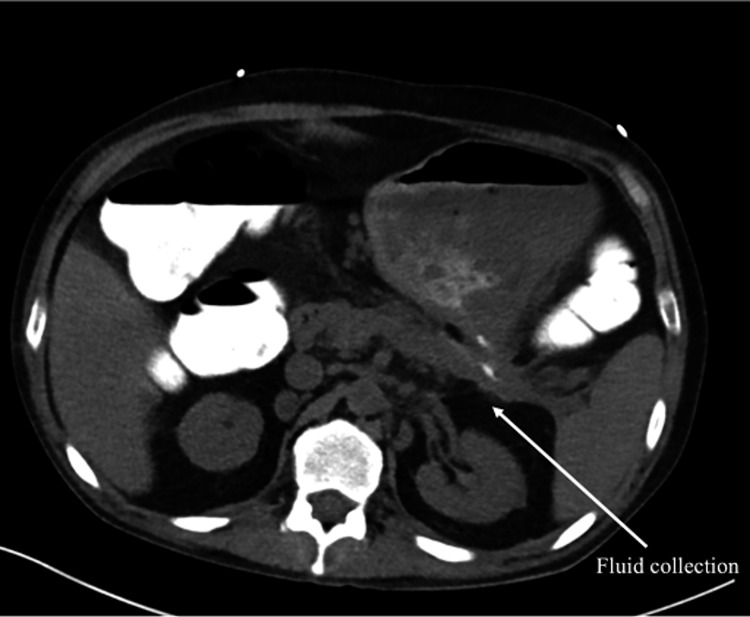
Axial CT scan with rectal contrast demonstrating extravasation of contrast (arrow) from the splenic flexure of the colon into a retroperitoneal inflammatory collection, confirming a pancreatico-colonic fistula

**Figure 2:**
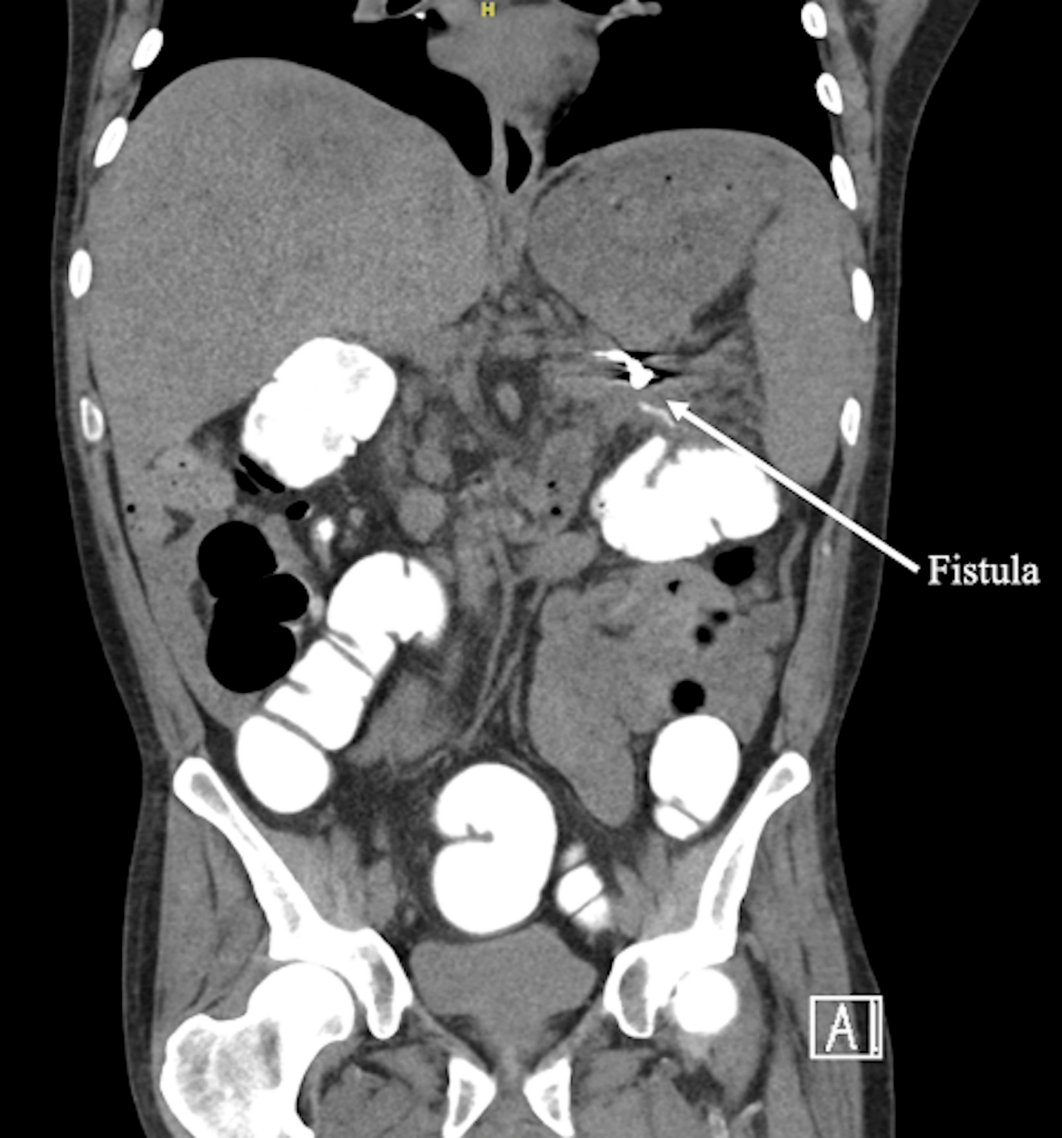
Sagittal CT scan with rectal contrast demonstrating extravasation of contrast (arrow) from the splenic flexure of the colon into the retroperitoneal inflammatory collection, confirming a pancreatico-colonic fistula

Five months later, the patient was admitted for hyperglycemia and ketonemia due to T2DM medication noncompliance and COVID-19, which resolved. Blood cultures drawn on the first day of hospital admission grew *Streptococcus viridans,* and repeat cultures continued to grow Streptococcus species. Possible causes of infection were explored, such as endocarditis due to poor dentition and septic arthritis due to a complaint of right-sided knee pain. All diagnostic tests for these infections, such as an echocardiogram and culture of joint aspirate, were negative. He was initially treated with Penicillin G on the floor, but was soon moved to the MICU and transitioned to vancomycin and meropenem due to persistent bacteremia. CT abdomen and pelvis with IV contrast showed a small left retroperitoneal fluid collection in the previously drained site (LLQ retroperitoneum). Air foci within the fluid collection and inflammatory stranding in the left retroperitoneal space were concerning for a fistulous connection to the adjacent large bowel. CT AB/P with rectal contrast showed extravasation of contrast into the fluid collection, confirming the fistulous connection at the splenic flexure to the inflammatory stranding within the left retroperitoneal space (Figures 1, 2). Colonoscopy showed erythema at the splenic flexure; no fistula sinuses were observed, and this area was tattooed. He was scheduled for a robotic laparoscopic left colectomy, takedown of splenic flexure, closure of pancreatico-colonic fistula, and end colostomy.

Therapeutic intervention

The pancreatico-colonic fistula was treated by robotic minimally invasive fistula takedown, partial colectomy, and end colostomy. Dissection of the fistula was done carefully due to dense adhesions and inflammatory changes in the peripancreatic retroperitoneum as well as significant bleeding from large collaterals and coagulopathy. The pancreatico-colonic fistula was identified and taken down using the vessel sealer, and the colon was separated with minor fecal spillage. The area of the pancreatic fistula was then closed using a running 2-0 prolene stratafix and buttressed with omentum. The splenic flexure was completely mobilized, and the descending colon was transected using a 45 mm Endo GIA stapler (Medtronic, Dublin, Ireland) with a green load. Colostomy creation instead of primary anastomosis was selected due to the patient’s malnourished state, extensive bleeding, and minor fecal spillage. After the abdomen was irrigated and hemostasis confirmed, the resected colon was removed through a suprapubic Pfannenstiel incision (sent to pathology), 2 Blake drains were placed (one around the spleen and tail of the pancreas and another in the pelvis), and the colostomy site was matured. Pathology report on the resected colon showed multiple perforations (0.2 -1.5cm diameter) with hemorrhage, granulation tissue, and fibrosis. A 0.2 cm tubular adenoma with low-grade dysplasia was also found with negative surgical margins.

Follow-up and outcomes

**Figure 3:**
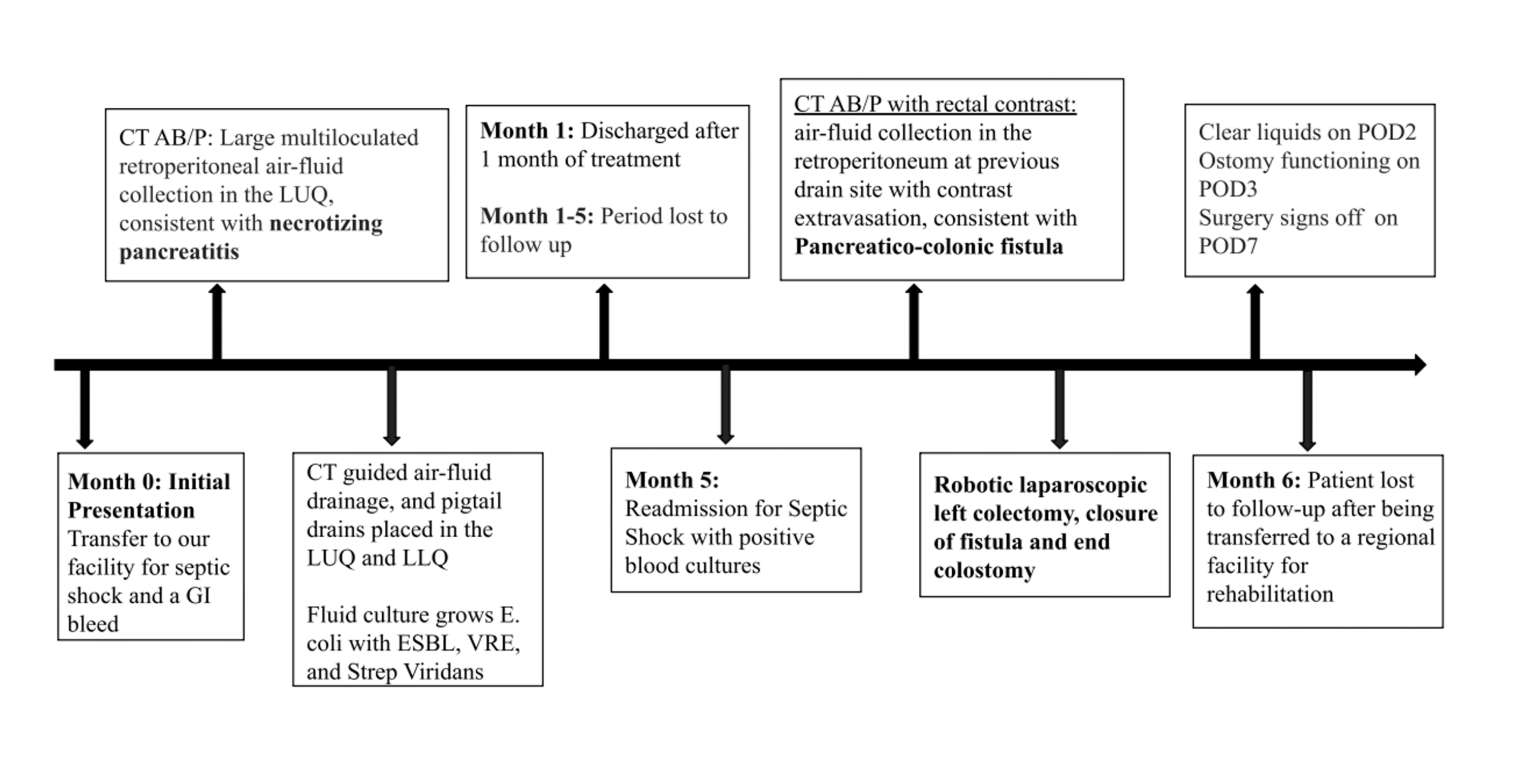
Timeline of case presentation ESBL: extended-spectrum beta-lactamases, VRE: vancomycin-resistant Enterococcus faecium, LUQ: left upper quadrant, LLQ: left lower quadrant, CT AB/P: computed tomography scan of the abdomen and pelvis; POD: postoperative day

After the procedure, the patient recovered well with adequate pain control. He was advanced to a clear liquid diet on postoperative day (POD) 2, and his ostomy functioned appropriately after the use of a laxative on POD 3. He noted marked improvement in abdominal pain postoperatively. Drains were removed on POD 7 after decreased output and CT AB/P with oral and IV contrast confirming a lack of re-accumulated abscess or fluid collection. The patient was successfully discharged from the surgery service on POD 7, but remained admitted under medicine for continued treatment of his infection. He was later transferred to a regional hospital for extensive rehabilitation. He did not follow up for colostomy reversal. Figure 3 shows the timeline of this case.

## Discussion

Acute pancreatitis is an inflammatory condition caused by the obstruction of pancreatic secretions [3]. Normally, the pancreas releases inactive lysosomal and lytic enzymes that are activated in the duodenum to aid digestion [3]. When outflow is obstructed, these enzymes become prematurely activated within the pancreas, leading to autodigestion of acinar cells and a local inflammatory response [3,7,8]. The resulting inflammatory fluid may accumulate as a pancreatic abscess or pseudocyst [3,9]. Spontaneous decompression of these collections releases activated enzymes into adjacent tissues, causing local tissue digestion and formation of fistulas with nearby viscera, including the stomach, duodenum, small intestine, colon, or biliary tree [5,6,9-11]. This process is driven by a combination of pressure necrosis from the fluid collection and enzymatic destruction of surrounding tissue [9,12,13].

Colonic involvement most commonly occurs at the transverse colon or splenic flexure, reflecting their proximity to the pancreas [8]. Compared with other pancreatico-enteric fistulas, colonic fistulas are particularly severe due to the high bacterial and fungal load of the colon [8,10,11]. Without prompt management, complications such as sepsis, hemorrhage, ileus, ischemia, necrosis, and perforation can occur [8,12]. Conservative management is associated with high mortality, and surgical resection of the affected colon, often with distal pancreatectomy and drainage of peripancreatic collections, remains the preferred approach [9,12]. A diverting ileostomy or colostomy is typically performed to allow inflammation to resolve before primary anastomosis [12]. Minimally invasive options, such as endoscopic fibrin-glue or hemoclip closure, may be effective for small fistulas (<10 mm) [10].

Approximately 60% of pancreatico-colonic fistulas present insidiously with nonspecific symptoms such as nausea, vomiting, hematochezia, or watery diarrhea [7,12]. Abdominal pain is common, often left-sided due to the proximity of the descending colon to the pancreas [9,12]. In a case series of patients undergoing surgery for severe acute pancreatitis, up to 41% developed fistulas, most frequently involving the colon and often forming postoperatively [12]. Table 1 compares this case presentation to other case reports on pancreatico-colonic fistulas.

**Table 1:** Comparison of reported pancreatico-colonic fistula cases

Author / Year	Etiology / Underlying Condition	Location of Fistula	Clinical Presentation	Diagnostic Modality	Management	Outcome
Present Case	Chronic necrotizing pancreatitis following gallstone pancreatitis; ESBL E. coli infection; poor follow-up	Splenic flexure of the colon	Septic shock, recurrent bacteremia, no typical GI symptoms	CT abdomen/pelvis with IV and rectal contrast confirming extravasation	Robotic-assisted laparoscopic left colectomy, takedown of fistula, and end colostomy	Improved post-operatively, discharged POD7; colostomy remained in place
Aryan et al., Am J Gastroenterol, 2022 [10]	Acute necrotizing pancreatitis with pseudocysts	Splenic flexure of the colon	Diffuse abdominal pain and loose stools	CT abdomen and pelvis with IV and oral contrast; colonoscopy confirming fistula opening	Endoscopic closure using an OTSC (Over-the-scope-clip)	Resolution of symptoms
Ghanimeh et al., Case Rep Gastrointest Med, 2018 [12]	Recurrent acute pancreatitis secondary to heavy alcohol use	Splenic flexure of the colon	Recurrent abdominal pain, fever, and diarrhea	CT abdomen and pelvis with IV and oral contrast; colonoscopy confirming fistula opening	Pancreatectomy, splenectomy, and partial left colectomy	Improvement post-operatively, with complete resolution of symptoms
Rodríguez et al., Gastroenterol Hepatol, 2016 [14]	Chronic pancreatitis with pseudocyst rupture	Descending colon	Sepsis, abdominal pain, diarrhea, malnutrition	CT and colonoscopy	Necrosectomy, Left colectomy with pancreatic drainage, cholecystectomy	Died of septic shock

The patient presented here did not have the expected symptoms during the admission in which the fistula was discovered. This is most likely due to the simultaneous presentation of diabetic ketoacidosis (DKA), COVID-19, and septicemia. However, persistent bacteremia after the resolution of DKA and COVID-19, coupled with a history of necrotizing pancreatitis with possibly inadequate hospital follow-up, raised suspicion for a persistent peripancreatic infection from his previous CT-guided drainage of fluid. The choice of surgical management for this patient was informed by the aforementioned standard of surgical management of pancreatico-colonic fistulas. Endoscopic closure of the fistula would not have been the best choice for this patient because, as per the pathology report, there were multiple perforations with a diameter of 1.5 cm in the greatest dimension. This less invasive procedure has been shown to be more effective in fistulas <10 mm in diameter [10]. Conservative management was not a good option either because this fistulation and surrounding inflammation were the only identified cause of bacteremia and sepsis. Other possible causes of infection were explored, such as endocarditis due to poor dentition and septic arthritis due to the complaint of right-sided knee pain. All diagnostic tests for these infections, such as an echocardiogram and culture of joint aspirate, were negative.

Key clinical takeaways

Although pancreatico-colonic fistulas are a seemingly uncommon complication, it is important to consider this diagnosis in patients with a history of pancreatic surgery, and especially in the case of severe/necrotizing pancreatitis.

Close follow-up post-pancreatic surgery/procedure is of utmost importance to detect possible pancreatico-enteric fistula formation before further complications and irreversible damage occur.

## Conclusions

Although rare, pancreatico-colonic fistulas have the potential to cause significant complications, especially in patients with a history of severe necrotizing pancreatitis, as seen in this case. Clinicians must stress the importance of proper post-surgical follow-up to these patients, as lack of postoperative follow-up likely contributed to the severity of this case. They must also maintain a high index of suspicion for such complications in patients who present with unconventional symptoms and a history of severe pancreatitis, such as this case. Surgical management continues to be the best course of treatment for larger pancreatico-enteric fistulas, as persistent infections are more than likely to cause severe complications if not treated appropriately.
